# Comparative Efficacy and Safety of Botulinum Toxin and Nitroglycerin in the Treatment of Chronic Anal Fissures: A Systematic Review and Meta‐Analysis of Clinical Trials

**DOI:** 10.1155/bmri/6831460

**Published:** 2026-08-28

**Authors:** Hamid Reihani, Ali Daneshmandi, Amir Hossein Hassani, Maryam Hashemi

**Affiliations:** ^1^ Student Research Committee, School of Medicine, Shiraz University of Medical Sciences, Shiraz, Iran, sums.ac.ir; ^2^ School of Medicine, Shiraz University of Medical Sciences, Shiraz, Iran, sums.ac.ir

**Keywords:** botulinum toxins, chronic anal fissures, nitroglycerin, Type A

## Abstract

**Background:**

Chronic anal fissures (CAF) are a common anorectal condition that significantly impacts patients′ quality of life. Treatment options include pharmacological therapies, such as botulinum toxin (BT) and topical nitroglycerin (TNG). Despite their widespread use, the relative efficacy and safety of BT and TNG remain controversial. This systematic review and meta‐analysis is aimed at comparing the effectiveness, recurrence rates, and adverse effects of BT and TNG in the treatment of CAF.

**Methods:**

A comprehensive search was conducted across multiple databases, including PubMed, Scopus, and Cochrane. Studies were included if they compared BT with TNG in the treatment of CAF. Ten clinical trials, comprising 698 patients, were included. Data were extracted and analyzed using STATA, and risk ratios were calculated for healing rates, recurrence rates, and side effects.

**Results:**

The analysis showed that BT resulted in significantly higher healing rates compared with TNG (risk ratio: 1.18, 95% CI: 1.02–1.36, *p* = 0.029). However, there was no significant difference in recurrence rates between the two treatments (*p* = 0.146). BT was associated with a significantly lower incidence of side effects, including headaches, compared with TNG (*p* < 0.001).

**Conclusion:**

BT is more effective than TNG in the healing of CAF, with a superior safety profile. These findings suggest that BT may be the preferable treatment option for CAF.

## 1. Introduction

Anal fissure is a painful anorectal condition that significantly affects the quality of life and often causes emotional and psychological distress [1]. This condition is characterized by a linear tear in the anal canal that begins just below the dentate line and extends to the anal margin. Anal fissures can be either acute or chronic [2]. Recurrent anal fissures or those that last more than 4 weeks are generally categorized as chronic. The existence of anal papillae, sentinel piles, indurated borders, and exposed sphincter muscle fibers at the fissure′s base are all characteristics of chronic anal fissures (CAF) [3]. Anal fissures affect males and females equally and are most commonly observed in middle‐aged individuals [4]. The pathophysiology is still debatable, but two elements are thought to be most important: internal anal sphincter hypertonicity and local ischemia [5–7]. As a result, the goal of therapeutic options is to lower sphincter tone and enhance blood flow with subsequent tissue repair, using either pharmacological drugs such as topical nitroglycerin (TNG), calcium channel blockers (CCB), and botulinum toxin (BT), or surgical interventions such as lateral internal sphincterotomy (LIS) [8].

LIS is considered the gold standard for surgical management of CAF when conservative and medical treatments are ineffective. However, there are postoperative concerns including surgical risks and the possibility of long‐term irreversible incontinence [3, 9, 10]. BT and TNG have become popular treatment alternatives due to their ability to lower anal sphincter tone and increase blood flow to the affected area [11, 12]. BT works by temporarily paralyzing the sphincter muscle, eliminating sphincter hypertonia, and raising local tissue perfusion, resulting in the healing of CAF [13]. TNG, on the other hand, operates as a vasodilator, boosting blood flow and decreasing anal pressure through its actions on the anal canal′s smooth muscle [5, 14–16].

Although BT and TNG are widely used for the treatment of CAF, their relative efficacy remains controversial. Existing studies report varying outcomes in terms of healing rates, pain reduction, and recurrence rates. Previous systematic reviews and meta‐analyses were limited by insufficient studies, the exclusion of articles in non‐English languages, and the omission of grey literature. This systematic review and meta‐analysis is aimed at providing a comprehensive comparison of BT and TNG, evaluating their effectiveness, recurrence rates, and adverse effects in the management of CAF.

## 2. Methods

### 2.1. Search Strategy

To ensure a rigorous and transparent approach, we conducted this systematic review and meta‐analysis of clinical trials in accordance with the guidelines outlined in the PRISMA (Preferred Reporting Items for Systematic Reviews and Meta‐Analyses) checklist. A comprehensive literature search was performed on March 24, 2025, to determine the most relevant keywords for the study. Based on these keywords, tailored search strategies were developed for each of the following databases: PubMed/Medline, Scopus, Cochrane Central Register of Controlled Trials (CENTRAL), Google Scholar, Web of Science (WOS), Magiran, Embase, and SID (in Persian). An example of the search syntax utilized for the PubMed database is provided below: (“Botulinum Toxins, Type A”[Mesh] OR “Botulinum Toxins, Type A” [Title/Abstract] OR “Botulinum Neurotoxin Type A” [Title/Abstract] OR “Botulinum” [Title/Abstract] OR “Botox” [Title/Abstract] OR “Neurotoxin” [Title/Abstract] OR “Oculinum” [Title/Abstract] OR “Neuronox” [Title/Abstract] OR “Onabotulinumtoxin” [Title/Abstract] OR “Clostridium” [Title/Abstract]) AND (“Nitroglycerin” [Mesh] OR “Nitroglycerin” [Title/Abstract] OR “Glyceryl Trinitrate” [Title/Abstract] OR “Nitro‐Dur” [Title/Abstract] OR “Sustac” [Title/Abstract] OR “Tridil” [Title/Abstract] OR “Trinitrin” [Title/Abstract] OR “Anginine” [Title/Abstract] OR “Gilustenon” [Title/Abstract] OR “Nitrangin” [Title/Abstract] OR “Nitro Bid” [Title/Abstract] OR “Perlinganit” [Title/Abstract] OR “Sustak” [Title/Abstract] OR “Nitrangin” [Title/Abstract]).

### 2.2. Protocol Registration

We have registered our review protocol on the Open Science Framework (OSF) to ensure transparency and reproducibility of our research process. The protocol can be accessed through the following link: 10.17605/OSF.IO/97QDH.

### 2.3. Eligibility Criteria

The eligibility criteria for inclusion in this study were as follows: (a) the study must be a clinical trial, and (b) the study must compare BT with nitroglycerin in the treatment of CAF. Studies focused on hemorrhoids were excluded. Additionally, studies that used isosorbide dinitrate were excluded too. There were no language or geographical restrictions, and grey literature was also included in the analysis. As this was a systematic review of published clinical trials, no personal patient information was included, and approval from the institutional ethics committee was not required.

### 2.4. Selection Process

The search results were exported in a format compatible with EndNote X7 reference management software, where they were organized accordingly. Duplicate studies were subsequently removed. Two independent reviewers (H.R. and M.H.) conducted the title and abstract screening, followed by a full‐text review of the selected studies. Any discrepancies during the review process were resolved through consensus. Additionally, the reference lists of the included studies were examined to identify any relevant articles that may have been missed during the initial search.

### 2.5. Data Collection

Two researchers (A.D. and A.H.H.) conducted a detailed review of the selected studies and extracted the following variables: first author, year of publication, study design, location (city, country, research institution), study objectives, sample size, type and dosage of intervention, healing rate, recurrence rate, adverse effects, and demographic characteristics of the participants.

### 2.6. Risk of Bias Assessment

The revised Cochrane risk‐of‐bias tool for randomized trials (RoB 2) was used to assess the risk of bias in included studies. H.R. and A.D. independently assessed the risk of bias in each study. M.H. supervised the assessment process and minor disagreements were solved through consensus.

### 2.7. Statistical Analysis

The collected data were analyzed using STATA Version 17.0 software and the metan package. Risk ratios (RRs) with 95% confidence intervals (CIs) were calculated for dichotomous outcomes (healing rate, recurrence rate, and side effects), and a random‐effects model was employed for meta‐analysis, accounting for potential heterogeneity. Heterogeneity was assessed with Cochran′s *Q* test and the *I* ^2^ statistic, whereas publication bias was evaluated using the Egger and Begg tests. All tests were two‐sided, with a significance level set at *p* < 0.05.

## 3. Results

### 3.1. Study Selection and Characteristics

**Figure 1:**
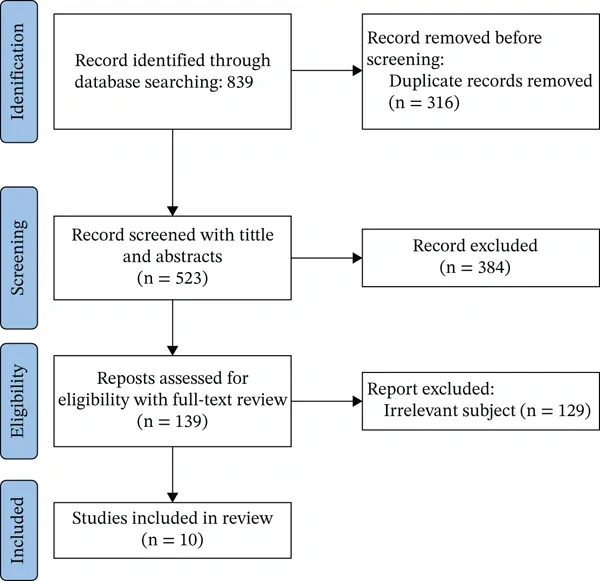
Preferred Reporting Items for Systematic Reviews and Meta‐Analyses (PRISMA) flowchart.

Eight hundred and thirty‐nine studies were identified according to the search criteria mentioned above. After removing duplicates, we proceeded to review a total of 523 publications. After reviewing titles, abstracts, and full texts, we found 10 publications that matched our set criteria for inclusion [17–26]. A total of 698 patients (348 in the BT group and 350 in the TNG group) were included in the final analysis. Figure 1 illustrates the selection process through a flowchart. The study characteristics are detailed in Table 1. Eight studies reported on healing rates after Botox injections versus TNG ointments; six of these also reported recurrence rates. Eight studies reported the prevalence of side effects, including headaches.

**Table 1:** Characteristics of included studies.

Author (year)	Country (language)	BT group	TNG group	Age	Total number (BT/TNG)	Healing rate (%)	Recurrence rate (%)	Side effects rate (%)	Follow‐up duration
Anari et al. (2021) [26]	Iran (English)	BOTOX	TNG	41.42	38 (19/19)	NR	BT group: 0, TNG group: 0	NR	5 weeks
		Ointment	Ointment						
		BID	BID						
		4 weeks	4 weeks						
Timilsina et al. (2018) [17]	Nepal (English)	30 U (15 U each side)	TNG 0.2% TID 8 weeks	31.23	112 (56/56)	BT: 89, TNG: 70	BT: 7.8, TNG: 25.4	BT: 0 TNG: 30	6 months
Gil et al. (2010) [18]	Spain (English)	25 U (12.5 each side)	TNG 0.2% TID 8 weeks	44.4	128 (63/65)	BT: 51, TNG: 54	NR	NR	NR
Abdelhady et al. (2009) [19]	Egypt (English)	40 U (20 U each side)	TNG 0.2% BID 4–6 weeks	34.4	80 (40/40)	NR	BT: 52.5, TNG: 57.5	BT: 7.5 TNG: 20	5 years
Brisinda et al. (2007) [20]	Italy (English)	30 U Botox or 90 U Dysport	TNG 0.2% TID 8 weeks	44.1	100 (50/50)	BT: 92, TNG: 70	BT: 0, TNG: 14	BT: 6 TNG: 34	BT: 21.2 months, TNG: 20.7 months (mean)
Fruehauf et al. (2006) [21]	Switzerland (English)	30 U (10 U each side and 10 U dorsal)	TNG 0.2% BID 2 weeks	38	50 (25/25)	BT: 24, TNG: 52	NR	BT: 4 TNG: 48	12 weeks
De Nardi et al. (2006) [22]	Italy (English)	40 U (20 U each side)	TNG 0.2% TID 8 weeks	41.8	30 (15/15)	BT: 33.3, TNG: 40	BT group: 0.333, TNG group: 0.333	BT group:0 TNG: 20	Ranged between 36 and 46 months
Uluutku et al. (2001) [23]	Turkey (Turkish)	25 U (12.5 U each side)	TNG 0.2% TID 4 weeks	36.5	50 (25/25)	BT: 83.3, TNG: 73.9	BT group: NR, TNG group: NR	BT: 4 TNG: 28	2 months
Salman and Dabestani (2001) [24]	Iran (Farsi)	80 U Dysport (40 U each side)	TNG 0.3% BID 4–8 weeks	35.6	60 (30/30)	BT: 90, TNG: 80	BT group: 0.074, TNG group: 0.166	BT: 3.3 TNG: 46.6	6 months
Brisinda et al. (1999) [25]	Italy (English)	20 U (10 U each side)	TNG 0.2% BID 6 weeks	42.15	50 (25/25)	BT: 96, TNG: 60	BT group: 0, TNG group: 0	BT: 0 TNG: 28	Mean BT: 15.4 ± 4.1 months, TNG: 16.1 ± 2.6 months

### 3.2. Overview of the Definitions of CAF as Presented in the Existing Literature

CAF was clearly defined in all studies except for three [18, 19, 24], which did not provide a precise definition of CAF. However, the studies interpreted CAF in varied ways depending on how it was diagnosed and how long it lasted. Although the majority of the cited research [20, 21, 23, 25] classified CAF as a condition lasting more than 2 months, De Nardi et al. [22] believed it to be more than 3 months, Timilsina et al. [17] defined it to be more than 6 weeks, and Anari et al. [26] did not specify the duration and were diagnosing CAF based on clinical assessment and surgical examination.

### 3.3. Intervention Applied in the Studies

Out of the 10 trials included in Table 1, all utilized BT injections, except for one study (Anari et al. [26]) that employed BT ointment. Regarding TNG, all studies used a 0.2% concentration, except for Salman and Dabestani [24], which used TNG 0.3%. The duration of TNG application typically ranged from 4 to 8 weeks, although Fruehauf et al. [21] conducted a 2‐week assessment. Half of the studies applied TNG ointment twice daily, whereas the other half applied it three times daily. Of the nine trials using injectable BT, all administered the injection bilaterally into the internal anal sphincter, whereas Fruehauf et al. [21] injected into three locations: two lateral and one dorsal to the fissure. The BT dosages ranged from 20 to 30 units.

### 3.4. Healing Rate

**Figure 2:**
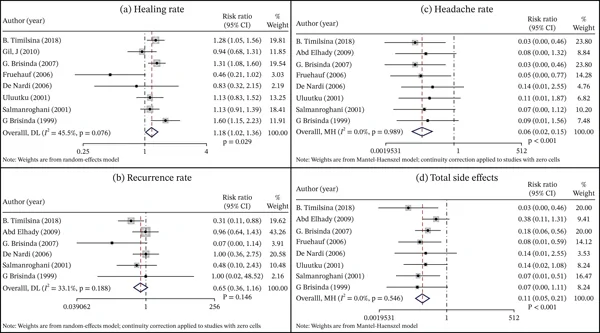
(a) The meta‐analysis of healing rates demonstrated a significant advantage of Botox over TNG. (b) No significant difference was observed in recurrence rates between the two groups. (c) The incidence of headaches was higher in the TNG group. (d) The overall rate of side effects was also greater in the TNG group.

With the exception of Anari et al. [26], who used BT ointment, and Abd elhady et al. [19], who did not specify the healing rate, all eight trials were included in the analysis comparing the fissure healing rates of BT versus the TNG group. The meta‐analysis demonstrated that patients treated with BT had significantly higher healing rates compared with those receiving TNG. The RR was 1.18, with a 95% CI ranging from 1.02 to 1.36 (*p* value: 0.029), indicating that BT‐treated patients had an 18% higher chance of healing than those in the TNG group. Moderate but insignificant heterogeneity (*I* ^2^ = 45.5*%*) was observed among studies (Figure 2). A sensitivity analysis revealed the study by Fruehauf et al. to be significantly influential, but the overall estimate remained significantly in favor of Botox when omitting this study.

### 3.5. Recurrence Rate

Pooling six studies reporting on recurrence rate, the RR was calculated as 0.65 (0.36–1.12). However, with a *p* value of 0.146, this difference did not achieve statistical significance, suggesting that there is insufficient evidence to conclude that BT is more effective than TNG in reducing recurrence rates (Figure 2). Heterogeneity was moderate (*I* ^2^ = 33.3*%*), and sensitivity analysis identified the study by Brisinda et al. to be significantly influential on the pooled estimate. After omitting this study, the overall recurrence rate was still comparable between the two groups.

### 3.6. Total Side Effects

An analysis of eight clinical trials assessing side effects revealed that participants who received BT had an 89% lower risk of experiencing side effects compared with those in the TNG group. The results were statistically significant (*p* < 0.001), with a RR of 0.11 (0.05–0.21) (Figure 2). Heterogeneity was negligible (*I* ^2^ = 0*%*), and sensitivity analysis found no outlier studies with significant influence on the overall estimate.

### 3.7. Headache Rate

Headache was the most commonly reported adverse effect, appearing in eight studies, and patients who received BT had a 94% lower risk of headaches than those in the TNG group (RR = 0.06, 95% CI = 0.02–0.15, *p* value < 0.001) (Figure 2). Heterogeneity was extremely low (*I* ^2^ = 0*%*), and sensitivity analysis found no studies with significant influence on the overall estimate.

### 3.8. Publication Bias

Egger′s test was employed to evaluate the potential for publication bias among the studies included in this analysis. The resulting *p* value of 0.112 suggests that there was no significant evidence of publication bias. However, given the limited number of studies included in this meta‐analysis, the results of the publication bias analyses should be interpreted with appropriate caution due to the limited statistical power of these tests.

### 3.9. Risk of Bias Assessment

**Figure 3:**
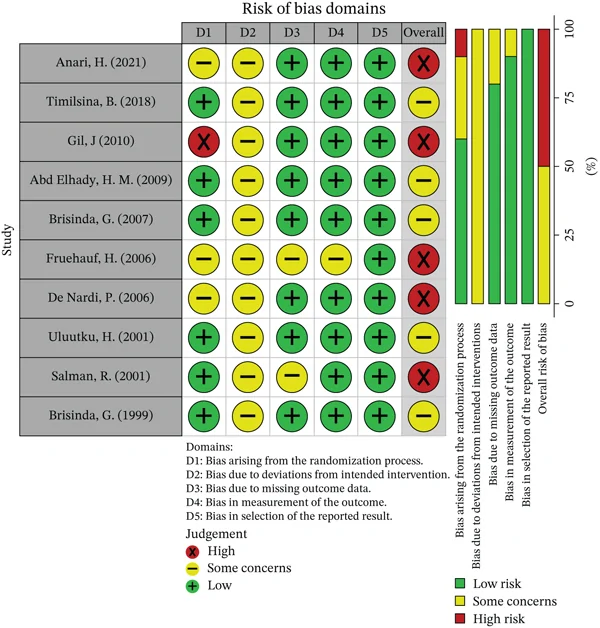
Risk of bias assessment across included studies, categorized by bias domain.

The risk of bias check showed that none of the studies included were rated as “low risk” overall (Figure 3) Most studies had “some concerns,” and several were considered “high risk.” Blinding was a major issue, likely because it is hard to hide whether patients received an injection (Botox) or an ointment (nitroglycerin), which helps explain why no studies achieved a low‐risk rating.

## 4. Discussion

This systematic review and meta‐analysis aimed at comprehensively evaluating the efficacy and safety of BT versus TNG in the treatment of CAF. Our findings demonstrate that BT significantly outperforms TNG in promoting healing, with a notable 18% higher ratio of healing. However, no significant difference was observed between the two treatments in terms of recurrence rates. Regarding side effects, BT showed a significantly lower risk of adverse events, including headaches, compared with TNG, highlighting its superior safety profile.

In our meta‐analysis, which included 10 studies, we found that BT significantly outperformed TNG in terms of healing rates and side effect profile. In contrast, the meta‐analysis by Sierra‐Arango et al. [27], which included only two studies comparing BT and nitroglycerin, found no statistically significant difference in healing rates between the two treatments, with a RR of 0.81 (95% CI: 0.02–29.36, *I* ^2^ = 93*%*). The difference in findings between our study and Sierra‐Arango et al. [27] can be attributed to the larger sample size in our analysis, whereas they provide a comparison between several topical agents used in the treatment of CAF, only two of the included studies exclusively compared BT and TNG. Our broader dataset provides more statistical power, enhancing the reliability of the results. Additionally, our inclusion of grey literature and non‐English studies reduced publication bias and offered a more comprehensive evaluation. The superior side effect profile of BT, as demonstrated in this study, is in agreement with Sierra‐Arango et al. [27] and existing literature.

Sahebally et al. [28] conducted a meta‐analysis of six studies, which revealed no statistically significant difference in healing and recurrence rates between BT and TNG for CAF. However, their research indicated that BT is associated with a decreased incidence of side effects, with RR = 0.12 (95% CI: 0.02–0.63, *p* = 0.01). In line with Sahebally et al. [28], our meta‐analysis found no significant difference in recurrence rates between BTX and TNG. Furthermore, our study demonstrated that BT increases the likelihood of healing by 18% compared with TNG. In addition, we found that the chance of side effects substantially decreases with BT, which is consistent with the findings of Sahebally et al. analysis. Furthermore, their analysis included studies that employed isosorbide dinitrate, which were excluded from our study, making our study more homogenous.

Compared with the meta‐analysis by Sajid et al. [29], which included three studies and found no significant difference in healing rates between BT and TGN (RR = 1.29, 95% CI: 0.98–1.70, *p* = 1.93), our study included 10 studies with a larger sample size and demonstrated a statistically significant superior healing rate with BT (RR = 1.18, 95% CI: 1.02–1.36, *p* = 0.029). Consistent with Sajid et al., our findings also demonstrate that BT is associated with fewer side effects compared with TGN. Sajid et al. reported a significantly lower incidence of side effects (RR = 0.14, 95% CI: 0.05–0.40, *p* = 0.0002) and headaches (RR = 0.07, 95% CI: 0.02–0.20, *p* = 0.0007) in the BT group.

This study has some limitations that should be considered prior to interpretation. First, considerable interstudy variability was observed in definitions (chronicity of disease and response to treatment) as well as dosing and administration of both agents. Additionally, the severity, sphincter pressure, and location of anal fissure remain unaddressed in the majority of studies. These factors could potentially influence the comparative effectiveness of local treatments, including Botox and TNG. The relatively short follow‐up durations also restrict the ability to assess long‐term outcomes effectively. Finally, risk of bias was assessed as moderate to high in the majority of studies, mainly due to randomization and blinding problems. To address these issues, future research should focus on standardizing CAF diagnostic criteria and extending follow‐up periods to better evaluate efficacy and recurrence. Additionally, we propose that future clinical trials should explore the combined use of TNG and BT, comparing this combination with each treatment used alone as well as to a placebo. This approach could provide valuable insights into the potential synergistic effects of these therapies in managing CAFs.

## 5. Conclusion

This review highlights that BT may be more effective than TNG in healing CAF, with a superior safety profile. Although both treatments showed similar recurrence rates, BT resulted in fewer side effects, particularly headaches. These findings suggest that BT could be considered a beneficial treatment option for CAF, though further large‐scale, high‐quality clinical trials are warranted to strengthen these conclusions.

## Funding

No funding was received for this manuscript.

## Consent

The authors have nothing to report.

## Conflicts of Interest

The authors declare no conflicts of interest.

## Data Availability

The datasets generated during the current study are available from the corresponding author on reasonable request.
